# EDUCATIONAL ATTAINMENT AND YOUNG ADULT HEALTH IN THE UNITED STATES: DIFFERENCES BY RACE AND GENDER

**DOI:** 10.1093/geroni/igz038.2194

**Published:** 2019-11-08

**Authors:** Grace A Noppert

**Affiliations:** University of North Carolina at Chapel Hill, Chapel Hill, North Carolina, United States

## Abstract

There is compelling evidence to suggest that educational disparities in health differ by both race and gender. This study examines the relationship between respondents’ education and six health outcomes related to cardiometabolic and inflammatory outcomes using data from Wave IV of the National Longitudinal Study of Adolescent to Adult Health (ages 24-32 years; N = 13,458). We used logistic regression models to examine the relationship between education and the odds of each health outcome. Models were stratified by race and gender. We found that the association between education and each health outcome differed by race/ethnicity and gender. While among whites we observed an association between education and each health outcome, for blacks we observed no such associations. It may be that the benefits of education are particularly salient for those in more structurally advantaged positions, pointing to the continued need to address structural inequalities by both gender and race.

